# KPC-2 Orchestrates Systemic Metabolic Rewiring to Facilitate Antibiotic Adaptation in *Klebsiella pneumoniae*

**DOI:** 10.3390/microorganisms14051054

**Published:** 2026-05-08

**Authors:** Peng Xie, Jing Sun, Xiangzheng Wu, Chenxi Liu, Luqi Li, Tong Wu, Yihan Luo, Caiyuan Zhou, Fang Wang, Yibin Hu, Leilei Sun, Chengbao Wang

**Affiliations:** 1 College of Veterinary Medicine, Northwest A&F University, Xianyang 712100, China; 2 Life Science Research Core Services, Northwest A&F University, Xianyang 712100, China

**Keywords:** KPC-2, *Klebsiella pneumoniae*, untargeted metabolomics, metabolic reprogramming, antibiotic stress, adaptive mechanism

## Abstract

The prevalence of *bla_KPC_*-carrying *Klebsiella pneumoniae* poses a significant clinical challenge, yet its systematic metabolic adaptations remain poorly understood. We employed an engineered *K. pneumoniae* (pACYC184/KPC-2) model and UHPLC-QE-Orbitrap-MS-based untargeted metabolomics to characterize metabolic shifts under steady-state and antibiotic-stressed conditions. OPLS-DA revealed profound metabolic divergence induced by KPC-2. At steady state, the 96 differential metabolites (30 up-regulated, 66 down-regulated) indicated enhanced cell membrane and nucleotide metabolism but suppressed carbohydrate and amino acid pathways. Under antibiotic challenge, the 90 differential metabolites (49 up-regulated, 41 down-regulated) indicated activated porphyrin, purine, and aromatic amino acid metabolism, alongside inhibited respiratory chain and lipid oxidative processes. NADH, FAD, and UDP were identified as core regulatory nodes. Our findings suggest that KPC-2 is associated with systematic metabolic rewiring that may facilitate bacterial survival and adaptation. These metabolic signatures provide novel insights into the putative physiological cost of resistance and potential therapeutic targets.

## 1. Introduction

The global escalation of antimicrobial resistance (AMR) has emerged as one of the most significant threats to public health in the 21st century. Among the ESKAPE (Enterococcus faecium, Staphylococcus aureus, Klebsiella pneumoniae, Acinetobacter baumannii, Pseudomonas aeruginosa, and Enterobacter species) pathogens, *Klebsiella pneumoniae* stands out as a primary culprit in hospital-acquired infections, ranging from pneumonia and bloodstream infections to neonatal sepsis [1]. The rapid dissemination of carbapenem-resistant *K. pneumoniae* (CRKP), primarily mediated by the production of carbapenemases such as the *Klebsiella pneumoniae* carbapenemase (KPC), has severely limited clinical treatment options and increased mortality rates worldwide [2].

While the genetic and molecular mechanisms of KPC-mediated resistance—centered on the enzymatic hydrolysis of carbapenems—are well-characterized, recent evidence suggests that antibiotic resistance is not an isolated genetic event [3]. Instead, it is a systemic process that imposes a significant physiological burden on the host cell. To offset the fitness costs associated with maintaining resistance genes and to ensure survival under antibiotic pressure, bacteria undergo profound “metabolic reprogramming” [4]. This metabolic plasticity allows pathogens to rewire their internal biochemical networks, optimizing energy production, precursor availability, and antioxidant defenses [5].

Despite the critical role of metabolism in bacterial physiology, the specific systematic metabolic alterations triggered by *bla*KPC gene expression remain under-explored [6]. Understanding these shifts is essential, as they may reveal “metabolic Achilles’ heels” that can be exploited to develop novel antibiotic adjuvants or therapeutic strategies.

In this study, we employed an engineered *K. pneumoniae* model (pACYC184/KPC-2) to isolate the metabolic contributions of the Klebsiella pneumoniae carbapenemase-2 (KPC-2) gene [7]. By utilizing high-resolution, untargeted metabolomics based on the UHPLC-QE-Orbitrap-MS platform, we performed a comprehensive analysis of the metabolic landscape in KPC-positive strains. We further investigated the metabolic responses of these strains under antibiotic challenge to identify key adaptive pathways [8]. Our findings reveal that KPC-2 expression orchestrates a complex rewiring of purine and pyrimidine metabolism, amino acid biosynthesis, and redox homeostasis, providing a systemic perspective on the survival strategies of this high-risk pathogen [9].

## 2. Materials and Methods

### 2.1. Bacterial Strains and Culture Conditions

The engineered strain was constructed by transforming the pACYC184-KPC-2 plasmid into *Klebsiella pneumoniae ATCC 13883*. The control strain harbored the empty pACYC184 vector. To maintain plasmid stability, all culture media were routinely supplemented with chloramphenicol (34 µg/mL) unless otherwise specified.

For metabolomics analysis, both strains were pre-cultured in Luria–Bertani (LB) broth at 37 °C with shaking at 200 rpm for 12 h. These pre-cultures were then diluted 1:100 in 50 mL of fresh LB broth in 250 mL conical flasks and incubated under identical conditions (37 °C, 200 rpm) until they reached the exponential phase (OD_600_ = 0.6–0.8). Six independent biological replicates were performed for each group, with each replicate derived from a separately inoculated overnight culture.

For antibiotic challenge experiments, KPC-2-carrying strains were exposed to a sub-inhibitory concentration of imipenem (0.5 × MIC) for 1 h prior to harvesting. The control strain (pACYC184) was processed in parallel under identical conditions to ensure comparability [10].

The pACYC184-based constitutive expression system was selected to ensure stable, uniform KPC-2 expression across all biological replicates, thereby minimizing the inter-sample metabolic variability associated with inducible promoter leakiness or heterogeneous induction kinetics.

The minimum inhibitory concentration (MIC) was predetermined by broth microdilution prior to metabolomics experiments, and culture growth was monitored spectrophotometrically as the optical density at 600 nm (OD_600_). Chloramphenicol at the plasmid maintenance concentration (34 µg/mL) did not measurably affect the growth rate or global metabolic profile of the empty-vector control strain relative to antibiotic-free cultivation. Both strains were harvested at equivalent OD_600_ values to ensure comparable physiological and fitness states at the time of metabolite extraction.

### 2.2. Sample Preparation and Metabolite Extraction

Bacterial cells were harvested during the exponential growth phase (OD_600_ = 0.6–0.8) by rapid centrifugation at 8000× *g* for 5 min at 4 °C. Metabolic activity was immediately quenched by resuspending the cell pellets in a pre-cooled methanol/ethylene glycol mixture (1:2, *v*/*v*) at −60 °C. The quenched suspensions were centrifuged at 8000× *g* for 5 min at −20 °C, and the resulting pellets were washed twice with ice-cold 0.85% NaCl to remove extracellular metabolites and residual medium components.

For metabolite extraction, the washed cell pellets were resuspended in boiling ethanol/water (75:25, *v*/*v*) and incubated at 95 °C for 10 min. Following extraction, the mixtures were centrifuged at 12,000× *g* for 10 min at 4 °C, and the supernatants were collected and dried under vacuum for subsequent UHPLC-QE-Orbitrap-MS analysis.

To monitor analytical reproducibility, quality control (QC) samples were prepared by pooling equal aliquots from all biological extracts and processing them alongside the experimental samples. Procedural blanks (extraction reagents without bacterial cells) were also included to exclude background contaminants [11].

### 2.3. UHPLC-QE-Orbitrap-MS Analysis

Metabolic profiling was performed using ultra-high-performance liquid chromatography–hybrid quadrupole–Orbitrap mass spectrometry (UHPLC-QE-Orbitrap-MS) on a quadrupole–Orbitrap mass spectrometer (Thermo Scientific, Waltham, MA, USA) coupled with both reversed-phase (RP) and hydrophilic interaction liquid chromatography (HILIC) separation techniques.

Chromatographic Separation: For RP separation, a BEH Shield RP C18 column (2.1 mm × 100 mm, 1.7 µm; Waters, Milford, MA, USA) was utilized. For HILIC separation, a BEH Amide column (100 mm × 2.1 mm, 1.7 µm; Waters, Milford, MA, USA) was employed. The flow rate was maintained at 0.3 mL/min with an injection volume of 2 µL [12].

Mass Spectrometry: The Orbitrap was operated in both positive and negative electrospray ionization (ESI) modes. The parameters included a capillary temperature of 300 °C, a spray voltage of 2.8 kV, and a resolution of 70,000. The scan range was set between 150 and 2000 m/z, utilizing stepped collision energies of 20, 40, and 60 eV [13].

### 2.4. Statistical Analysis

Metabolomic data were analyzed using SIMCA-P software (version 14.1, Umetrics, Umeå, Sweden). Orthogonal partial least squares-discriminant analysis (OPLS-DA) was employed to discriminate between the groups’ metabolic profiles. Differential metabolites were selected based on the following criteria: variable importance in projection (VIP) > 1.0, absolute log_2_ fold change (|log_2_FC|) > 1.0, and statistical significance based on Student’s *t*-test (*p* < 0.05). All statistical models were constructed using six biological replicates per experimental group.

## 3. Results

### 3.1. Reprogramming of Metabolic Profiles in K. pneumoniae Carrying the KPC Gene

**Figure 1:**
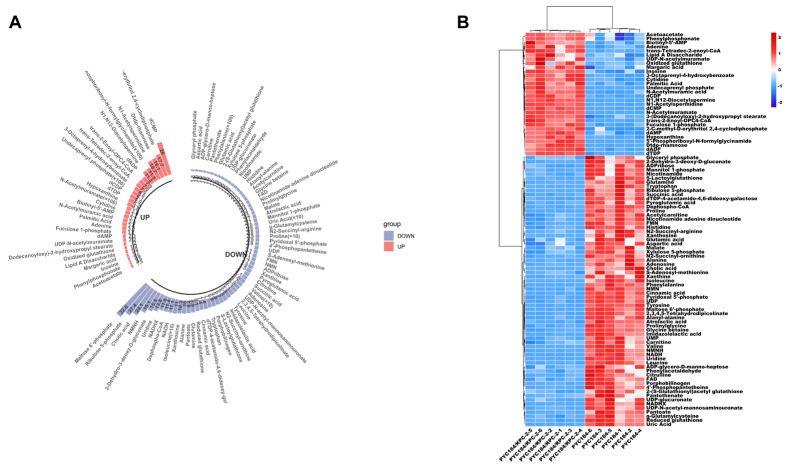
Reprogramming of metabolic profile of *Klebsiella pneumoniae* carrying KPC gene. (**A**) Up-regulation and down-regulation of differential metabolites in *Klebsiella pneumoniae* carrying the KPC gene. A total of 96 differential metabolites were identified, including 30 up-regulated and 66 down-regulated metabolites. The up-regulated metabolites mainly included 5′-phosphoribosyl-N-formylglycinamide, undecaprenyl phosphate, N-acetylmuramic acid, deoxycytidine monophosphate, 2-C-methyl-D-erythritol 2,4-cyclodiphosphate (an intermediate of the MEP pathway), and N-acetylspermidine. The down-regulated metabolites mainly included phenylalanine, isoleucine, valine, maltose-6′-phosphate, proline, uric acid, and cholic acid. (**B**) Heatmap analysis of differential metabolic profiles in *Klebsiella pneumoniae* carrying the KPC gene. Each column represents a treatment condition, and each row represents a metabolite. The normalized metabolite variation ranged from −2 (blue) to 2 (red). The top clustering tree showed the sample clustering relationship; samples of the two strains formed distinct independent clusters, indicating significant differences in metabolic profiles. All metabolic profiling data were derived from six independent biological replicates (*n* = 6) per group.

To rigorously evaluate the metabolic perturbations specifically elicited by the expression of the *bla_KPC-2_* gene, we established a comparative research model utilizing an engineered *Klebsiella pneumoniae* strain (pACYC184/KPC-2) alongside its corresponding control strain (pACYC184). The global intracellular metabolic landscape was captured using high-resolution UHPLC-QE-Orbitrap-MS, ensuring high sensitivity and broad coverage of the bacterial metabolome. To extract meaningful biological insights from the high-dimensional data, multivariate statistical modeling was performed. Orthogonal partial least squares-discriminant analysis (OPLS-DA) revealed a profound and highly significant metabolic divergence between the KPC-2-carrying group and the control group, indicating that the acquisition of this resistance gene fundamentally alters the host’s biochemical state. A comprehensive screening identified a distinct panel of 96 differential metabolites that met the strict selection criteria for significance. Within this metabolic signature, 30 species were significantly up-regulated, while 66 species exhibited marked down-regulation in the KPC-positive strain (Figure 1A). A functional enrichment analysis of the up-regulated pool revealed a strategic prioritization of cell envelope biogenesis and nucleotide turnover. Notably, key structural precursors such as undecaprenyl phosphate and N-acetylmuramic acid—essential components for peptidoglycan and cell wall assembly—showed substantial accumulation. Furthermore, intermediates of the non-mevalonate (MEP) pathway, specifically 2-C-methyl-D-erythritol 2,4-cyclodiphosphate, were elevated, suggesting an intensified investment in isoprenoid metabolism to enhance membrane integrity. Nucleotide metabolism was also significantly active, as evidenced by the accumulation of 5′-phosphoribosyl-N-formylglycinamide and deoxycytidine monophosphate (dCMP), which likely supported DNA repair and rapid signaling requirements. Conversely, the down-regulated metabolite pool was heavily dominated by primary growth-related nutrients, including specific amino acids, carbohydrates, and critical redox cofactors. The levels of branched-chain amino acids (valine and isoleucine) and aromatic amino acids (phenylalanine) were significantly depleted, alongside a reduction in maltose 6′-phosphate and proline. This profound suppression of core metabolic routes is consistent with a putative “fitness cost,” whereby the bacteria may strategically restrict carbohydrate utilization and protein synthesis to conserve energy for resistance-related processes.

To further validate the robustness of these findings, Hierarchical Clustering Analysis (HCA) was employed to visualize the metabolic fingerprints. As depicted in the heatmap in Figure 1B, the six independent biological samples were partitioned into two distinct, non-overlapping clusters corresponding to their genetic background. The high degree of similarity within each group and the sharp contrast between the two cohorts demonstrate the consistency and reproducibility of the metabolic shifts dictated by KPC-2. Collectively, these results confirm that KPC-2 expression does not merely facilitate antibiotic hydrolysis but orchestrates a comprehensive and systematic reconfiguration of the *K. pneumoniae* metabolic landscape to balance survival and physiological homeostasis.

### 3.2. Adaptive Metabolic Reprogramming of KPC-2-Carrying K. pneumoniae Under Antibiotic Challenge

**Figure 2:**
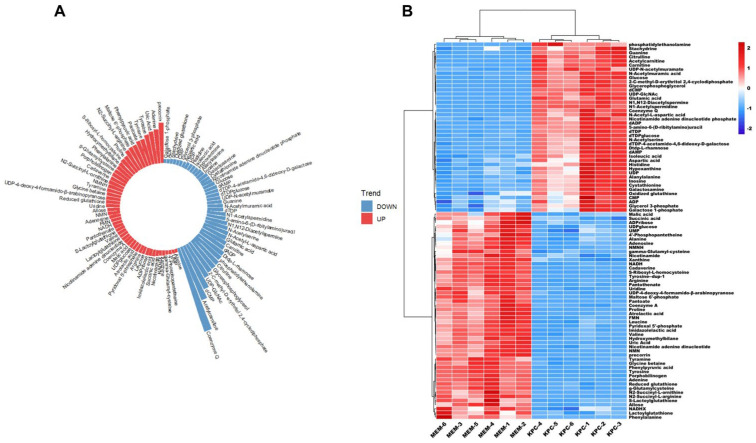
Heat map analysis of differential metabolic profiles of *Klebsiella pneumoniae* carrying KPC gene. (**A**) Up-regulation and down-regulation of differential metabolites in KPC gene-carrying *Klebsiella pneumoniae* under drug stimulation. A total of 90 differential metabolites were identified, among which, 49 were up-regulated and 41 were down-regulated. The up-regulated metabolites mainly included precorrin, adenine, uric acid, tyrosine, pantoyl lactone, phenylpyruvic acid, etc. The down-regulated metabolites mainly included coenzyme Q, acetylcarnitine, deoxycytidine monophosphate, uridine diphosphate-N-acetylglucosamine, citrulline, and phosphatidylethanolamine. (**B**) Heatmap analysis of differential metabolic profiles in KPC gene-carrying *Klebsiella pneumoniae*. Each column represents the treatment condition, and each row represents a metabolite. The variation range of normalized metabolites was from −2 (blue) to 2 (red). The top clustering tree showed the sample clustering relationship; samples of the two strains formed distinct independent clusters, indicating significant differences in metabolic profiles. All metabolic profiling data were derived from six independent biological replicates (*n* = 6) per group.

To investigate the metabolic mechanisms underlying antibiotic resistance, we analyzed the metabolic response of KPC-2-carrying *K. pneumoniae* under antibiotic stimulation. OPLS-DA modeling revealed significant clustering and separation between the resistant and control groups, indicating a distinct metabolic shift triggered by the drug challenge. A total of 90 differential metabolites were identified, with 49 significantly up-regulated and 41 down-regulated in the KPC-2-positive strain (Figure 2A).

The up-regulated metabolic pool was significantly enriched with precursors for porphyrin and purine metabolism, including precorrin, adenine, and uric acid, alongside aromatic amino acids such as tyrosine and phenylpyruvic acid. In contrast, the down-regulated metabolites were predominantly associated with cellular respiration and lipid metabolism, such as coenzyme Q, acetylcarnitine, and phosphatidylethanolamine. Notably, nucleotide-related species like dCMP and UDP-N-acetylglucosamine also exhibited a marked decrease under stress. These findings suggest that under antibiotic pressure, KPC-2-carrying bacteria prioritize the activation of porphyrin and purine biosynthesis to facilitate stress adaptation, while simultaneously suppressing respiratory chain functions and lipid oxidative processes to potentially reduce the production of metabolic by-products like reactive oxygen species (ROS).

Consistent with these observations, Hierarchical Clustering Analysis (HCA) demonstrated that the samples under antibiotic challenge formed two distinct lineages (Figure 2B). The heatmap revealed that the abundance of specific metabolites underwent systematic changes compared to the steady-state profiles. This coordinated transition in energy metabolism and biosynthetic pathways underscores the complex adaptive strategy employed by *bla_KPC-2_*-positive *K. pneumoniae* to survive in chemically hostile environments.

### 3.3. KPC-Mediated Perturbations in Key Metabolic Pathways Under Steady-State Conditions

**Figure 3:**
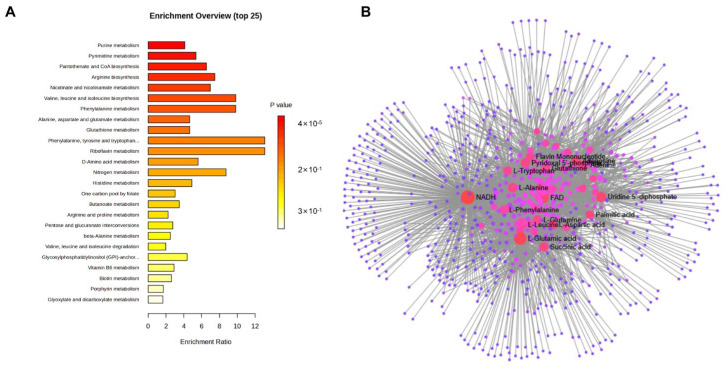
Systemic analysis of metabolic pathways and networks regulated by KPC genes. (**A**) KEGG pathway enrichment analysis displaying the most significantly altered metabolic routes in KPC-2-expressing strains. (**B**) Metabolite–metabolite interaction network analysis identifying core regulatory nodes based on topological importance. Node size is proportional to topological centrality, and node color indicates the centrality level (red, core regulatory hubs; pink, intermediate nodes; purple, peripheral nodes). All metabolic profiling data were derived from six independent biological replicates (*n* = 6) per group.

To transcend the identification of discrete metabolites and elucidate the underlying biochemical logic of KPC-induced reprogramming, we mapped the differential metabolic signatures onto the Kyoto Encyclopedia of Genes and Genomes (KEGG) database to perform pathway enrichment analysis (Figure 3A). This systemic approach revealed that KPC-2 expression does not act on isolated reactions but triggers a coordinated shift across multiple essential metabolic cascades. Compared to the pACYC184 control, the KPC-2-carrying strain exhibited significant enrichment in pathways critical for bacterial survival, including purine and pyrimidine metabolism, pantothenate and CoA biosynthesis, arginine metabolism, and the biosynthesis of branched-chain and aromatic amino acids. To pinpoint the “master regulators” of this network, we performed a metabolic topological analysis (Figure 3B), which identified reduced nicotinamide adenine dinucleotide (NADH), flavin adenine dinucleotide (FAD), and uridine diphosphate (UDP) as the core regulatory nodes. While these molecules are inherently central to bacterial metabolism as universal energy and redox currencies, their differential abundance and topological centrality in the KPC-2 network suggest that KPC-2 expression may perturb the flux equilibrium at these critical junctions, rather than creating novel regulatory roles [14].

### 3.4. Granular Dissection of Metabolic Perturbations and Adaptive Strategies

**Figure 4:**
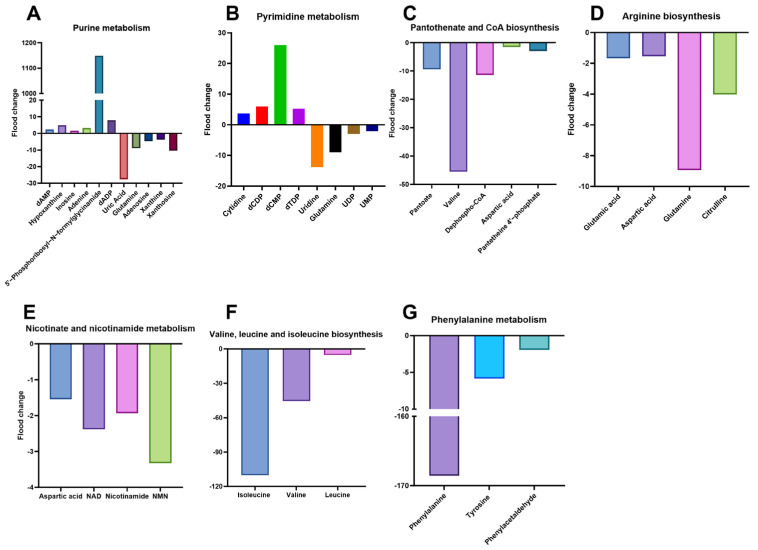
Specific metabolic perturbations in key biochemical pathways. (**A**) Metabolic perturbation of key metabolites in purine metabolism. (**B**) Metabolic perturbation of key metabolites in pyrimidine metabolism. (**C**) Metabolic perturbation of key metabolites in pantothenate and CoA biosynthesis. (**D**) Metabolic perturbation of key metabolites in arginine biosynthesis. (**E**) Metabolic perturbation of key metabolites in nicotinate and nicotinamide metabolism. (**F**) Metabolic perturbation of key metabolites in valine, leucine, and isoleucine biosynthesis. (**G**) Metabolic perturbation of key metabolites in phenylalanine metabolism. Metabolic perturbations depicted in this schematic diagram were identified from six independent biological replicates (*n* = 6) via OPLS-DA modeling (VIP > 1.0, |log_2_FC| > 1.0, *p* < 0.05 (Student’s *t*-test)). The directional arrows indicate statistically significant fold changes rather than absolute quantitative distributions; as such, standard deviation bars are not incorporated in this pathway-oriented visualization.

A granular examination of these individual biochemical routes (Figure 4A–G) provided deeper insights into the adaptive strategies employed by the pathogen. Regarding nucleotide metabolism and nitrogen allocation, we observed a massive accumulation of 5′-phosphoribosyl-N-formylglycinamide—a key intermediate in de novo synthesis—while terminal degradation products like uric acid and xanthosine were markedly depleted. This shift, coupled with the significant reduction in glutamine (the primary nitrogen donor), suggests that KPC-2-positive bacteria prioritize the conservation of nucleotide precursors over their catabolic breakdown. Similarly, regarding pyrimidine metabolism, there was a sharp elevation in deoxycytidine monophosphate (dCMP) levels, indicating a robust investment in DNA-related precursors even as uridine and glutamine pools were exhausted.

The KPC-2 metabolic phenotype was also characterized by cofactor limitations and amino acid scarcity. The biosynthesis of CoA, a universal acyl carrier essential for the TCA cycle and fatty acid metabolism, appeared to be severely bottlenecked; levels of the precursor pantoate and the intermediate dephospho-CoA were significantly reduced, alongside a drastic drop in valine, which serves as a carbon skeleton for pantothenate. Arginine biosynthesis, a crucial pathway for nitrogen homeostasis, also showed systemic down-regulation, with key precursors such as glutamate, aspartate, and citrulline showing varying degrees of depletion.

Furthermore, the subtle yet consistent reduction in NADH, nicotinamide, and nicotinamide mononucleotide (NMN) concentrations points to a perturbed redox environment, likely reflecting an increased demand for reducing equivalents to support resistance mechanisms. Finally, the biosynthesis of essential proteogenic amino acids was the most profoundly affected sector; the severe suppression of valine, isoleucine, and phenylalanine pools underscores a significant “biosynthetic drain,” where primary growth resources are sacrificed to sustain the metabolic burden imposed by the KPC-2 gene.

### 3.5. Systemic Metabolic Landscape of KPC-2-Carrying K. pneumoniae Under Antibiotic Stress

**Figure 5:**
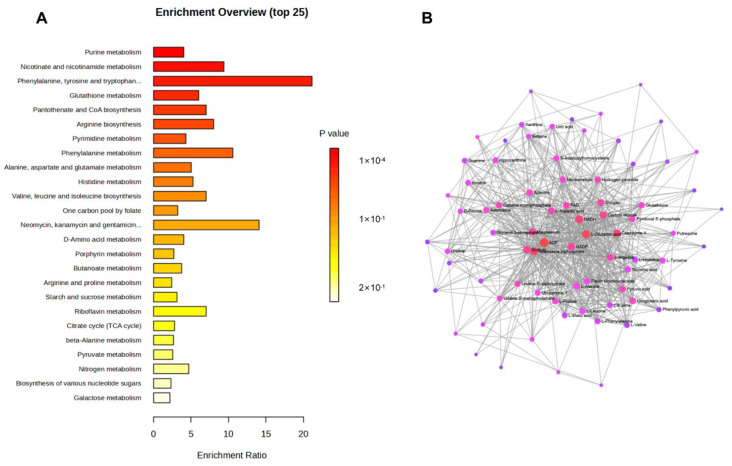
Systemic metabolic pathway and network analysis under antibiotic stress. (**A**) KEGG pathway enrichment analysis illustrating the most significantly altered metabolic routes in KPC-2-carrying strains during antibiotic challenge. (**B**) Metabolite–metabolite interaction network analysis identifying NADH, ADP, and L-glutamic acid as the central regulatory hubs under stress conditions. Node size is proportional to topological centrality, and node color indicates the centrality level (red, central regulatory hubs; pink, intermediate nodes; purple, peripheral nodes). All metabolic profiling data were derived from six independent biological replicates (*n* = 6) per group.

Under antibiotic challenge, the KPC-2-carrying strain demonstrated a distinct and complex metabolic reconfiguration that stood in sharp contrast to its steady-state profile. To capture this global shift, we utilized KEGG enrichment analysis, which highlighted significant perturbations in glutathione metabolism, purine and pyrimidine metabolism, and the biosynthesis of aromatic amino acids and CoA cofactors (Figure 5A). To further decode the regulatory architecture of this stress response, we performed a metabolite–metabolite interaction network analysis (Figure 5B). Within this stress-responsive network, NADH, adenosine diphosphate (ADP), and L-glutamic acid emerged as the central regulatory hubs, functioning as the primary mediators that coordinate the bacterial response to external drug pressure [15].

### 3.6. Granular Dissection of Stress-Induced Biochemical Shifts and Adaptive Responses

**Figure 6:**
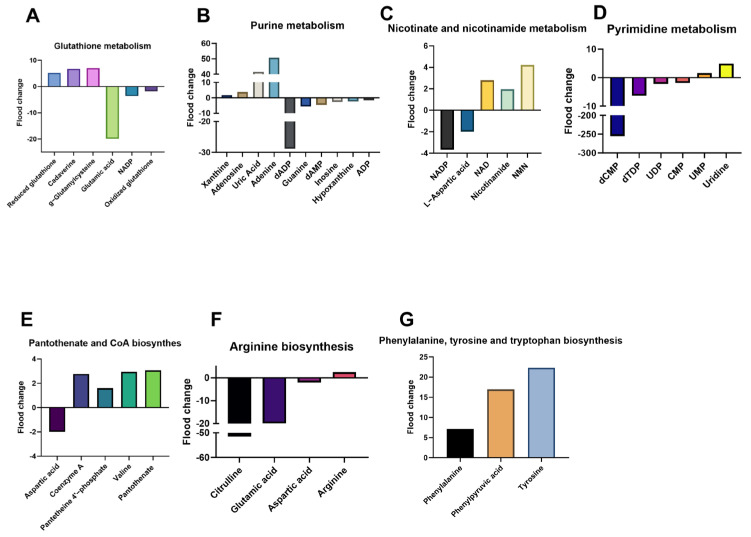
Specific metabolic perturbations in key biochemical pathways under antibiotic stress. (**A**) Metabolic perturbation of key metabolites in glutathione metabolism. (**B**) Metabolic perturbation of key metabolites in purine metabolism. (**C**) Metabolic perturbation of key metabolites in nicotinate and nicotinamide metabolism. (**D**) Metabolic perturbation of key metabolites in phenylalanine, tyrosine, and tryptophan biosynthesis. (**E**) Metabolic perturbation of key metabolites in pantothenate and CoA biosynthesis. (**F**) Metabolic perturbation of key metabolites in arginine biosynthesis. (**G**) Metabolic perturbation of key metabolites in pyrimidine metabolism. Stress-induced metabolic shifts shown herein were derived from six biological replicates (*n* = 6) per group. This figure presents a schematic overview of antibiotic-responsive pathways; individual metabolite fold changes met the stringent statistical thresholds of VIP > 1.0 and *p* < 0.05 (Student’s *t*-test). Standard deviation bars are omitted to preserve the clarity of the pathway-level illustration.

A detailed examination of specific metabolic sectors revealed the precise mechanisms through which the KPC-2 strain adapts to antibiotic-induced damage (Figure 6A–G). The antioxidant defense system appeared highly active; glutathione levels were significantly reduced while concentrations of γ-glutamylcysteine and cadaverine increased, suggesting an intensified demand on the cellular machinery to mitigate oxidative stress. This was accompanied by a notable shift in the redox environment, as evidenced by perturbations in nicotinate and nicotinamide metabolism. The nucleotide metabolic profile under stress underwent a complete reversal compared to steady-state conditions. Purine metabolism was marked by a significant accumulation of uric acid and adenine, whereas levels of dADP and dAMP were reduced. Similarly, in the pyrimidine pathway, dCMP levels—which were previously elevated in the absence of drugs—underwent a highly significant reduction during antibiotic challenge, indicating a shift from precursor accumulation to possible utilization or degradation under pressure. In contrast to the general scarcity observed under steady-state conditions, aromatic amino acid and cofactor pathways showed significant up-regulation. There was a consistent increase in the levels of phenylalanine, phenylpyruvate, and tyrosine, alongside elevated levels of CoA, pantetheine 4′-phosphate, and pantothenate. This up-regulation likely serves to meet the increased metabolic demands of stress adaptation and membrane repair. However, this recovery was selective, as significant decreases remained in citrulline, glutamate, and isoleucine levels, underscoring a strategic reallocation of nitrogen and carbon resources to prioritize survival mechanisms over primary growth.

### 3.7. Mechanistic Insight into KPC-2-Induced Global Metabolic Reprogramming and Associated Fitness Costs

**Figure 7:**
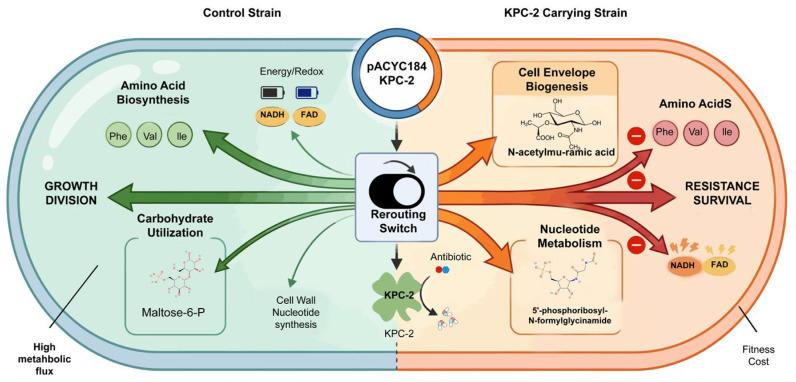
Adaptive resource allocation trade-offs in KPC-2-mediated metabolic redirection.

The acquisition and expression of the KPC-2 carbapenemase necessitate a profound systemic realignment of the Klebsiella pneumoniae metabolic landscape, characterized by a strategic reallocation of cellular resources from vegetative growth to defensive survival. Under homeostatic conditions, the metabolic flux of the control strain is optimized for maximal proliferative capacity, funneling carbon and nitrogen precursors primarily into anabolic pathways such as amino acid biosynthesis—specifically the synthesis of the branched-chain and aromatic amino acids phenylalanine, valine, and isoleucine—and efficient carbohydrate utilization via maltose-6-phosphate. This steady-state configuration maintains high intracellular energy charges and robust redox pools, ensuring a consistent supply of NADH and FAD to drive biomass accumulation. However, the introduction of the pACYC184-KPC-2 plasmid serves as a pivotal metabolic “trigger” that flips a central regulatory switch; beyond its primary role in antibiotic hydrolysis, KPC-2 expression induces a global rerouting of metabolic flux. In this adaptive state, the cell prioritizes the fortification of the cell envelope through the MEP pathway—synthesizing critical precursors like undecaprenyl phosphate and N-acetylmuramic acid—and the up-regulation of nucleotide metabolism (notably dCMP and 5′-phosphoribosyl-N-formylglycinamide) to support stress-induced signaling and DNA repair mechanisms. This strategic diversion of resources to “resistance and survival” occurs at the direct expense of primary metabolism, resulting in a significant attenuation of growth-promoting pathways and a precipitous exhaustion of the energy/redox pool. Consequently, the observed depletion of essential amino acids and the fluctuating energetic state of the cell provide a mechanistic basis for the fitness cost associated with KPC-2-mediated resistance, illustrating a fundamental evolutionary trade-off where metabolic efficiency is sacrificed for immediate physiological resilience (Figure 7).

## 4. Discussion

### 4.1. The Systemic Nature of KPC-2 Mediated Metabolic Rewiring

The global dissemination of carbapenem-resistant Enterobacteriaceae (CRE), particularly *Klebsiella pneumoniae* harboring the *bla_KPC-2_* gene, represents a critical challenge to modern medicine [16]. While traditional resistance research focuses on the enzymatic hydrolysis of carbapenems, our study reveals that KPC-2 expression triggers a profound and systemic reconfiguration of the bacterial metabolic landscape. This “metabolic reprogramming” is not a random collection of biochemical fluctuations but a coordinated adaptive response aimed at sustaining physiological homeostasis under the metabolic burden of resistance [17]. Our identification of 96 and 90 differential metabolites under steady-state and antibiotic-stressed conditions, respectively, underscores the complexity of this internal remodeling. It is important to note that the engineered pACYC184-KPC-2 model employed here drives constitutive, high-level KPC-2 expression to ensure experimental uniformity and robust metabolomic profiling. While this approach enables sensitive detection of metabolic shifts, it may not fully recapitulate the native, potentially regulated expression of *bla_KPC-2_* observed in clinical isolates. Consequently, the observed metabolic perturbations may partially reflect the physiological burden of plasmid maintenance and gene overexpression, rather than solely the direct metabolic integration of KPC-2 at intrinsic expression levels. Moreover, the generalizability of these findings to other K. pneumoniae genetic backgrounds requires independent validation. Future studies utilizing chromosomally integrated *bla_KPC-2_* constructs or tightly regulated inducible systems (e.g., arabinose- or IPTP-inducible) will be essential to disentangle KPC-2-specific metabolic effects from plasmid-associated fitness costs and to confirm the reproducibility of these metabolic signatures across diverse host strains.

An inherent limitation of our model is that the observed metabolic perturbations may partially reflect non-specific plasmid burden effects, including the energetic cost of plasmid replication and antibiotic selection pressure. While the empty-vector control (pACYC184) was employed to account for baseline plasmid effects, distinguishing KPC-2-specific metabolic integration from general plasmid-induced stress will ideally require a KPC-2 catalytic mutant or a complemented KPC clinical isolate in future studies.

### 4.2. Bioenergetic Trade-Offs and the Fitness Cost of Resistance

While the metabolic signatures observed here are highly suggestive of fitness trade-offs and resource reallocation, we acknowledge that these interpretations are inferred from untargeted metabolomic snapshots rather than directly demonstrated through functional phenotypic assays (e.g., competition experiments, isotopic tracing, or targeted envelope permeability assays). As such, the proposed “fitness cost” and “cell envelope fortification” should be regarded as testable hypotheses that warrant direct experimental validation.

A central theme in bacterial evolution is the “fitness cost” associated with the acquisition of resistance genes. Under steady-state conditions, KPC-2-carrying *K. pneumoniae* exhibited a significant down-regulation of primary metabolic pathways, including the metabolism of branched-chain amino acids (valine and isoleucine) and aromatic amino acids (phenylalanine) [18]. This suppression suggests a strategic resource reallocation, where the bacteria prioritize the synthesis of essential defense components at the expense of rapid growth [19]. Notably, the up-regulation of cell membrane precursors such as undecaprenyl phosphate and N-acetylmuramic acid indicates an intensified investment in cell wall and membrane biogenesis. This structural fortification likely serves as a passive defense mechanism to reduce the influx of antimicrobial agents [20]. The activation of the MEP pathway intermediate, 2-C-methyl-D-erythritol-2,4-cyclodiphosphate, further supports the hypothesis that KPC-2-positive strains reorganize their isoprenoid metabolism to enhance envelope integrity [16].

### 4.3. Nucleotide Metabolism: A Conserved Adaptive Strategy

Our pathway enrichment analysis highlighted purine and pyrimidine metabolism as central hubs of KPC-mediated reprogramming. Interestingly, the behavior of specific nucleotides like dCMP was highly context-dependent, showing a significant elevation under steady-state conditions but a sharp decline under antibiotic stress. This metabolic threshold suggests that bacteria modulate their nucleotide pools to balance DNA repair requirements with the need to limit metabolic byproducts. In our previous investigation into *tet*(X3)-mediated resistance, adenosine was identified as a critical metabolic biomarker that, when supplemented exogenously, could restore antibiotic sensitivity by inducing lethal oxidative stress [11]. In the current KPC-2 model, the fluctuations in 5′-phosphoribosyl-N-formylglycinamide and uric acid levels point to a similar vulnerability in purine turnover. These findings reinforce the notion that nucleotide metabolism represents a potentially conserved metabolic vulnerability across different resistance genotypes. However, in the KPC-2 context, this remains a speculative inference; whether exogenous purine derivatives (e.g., adenosine and inosine) can resensitize KPC-2-producing strains to carbapenems—analogous to their efficacy in *tet*(X3)-positive E. coli—constitutes an exciting but untested hypothesis that we propose as a priority for future experimental investigation [21].

### 4.4. Redox Homeostasis and the Transition to Stress-Adaptive Modes

Antibiotic challenge forces resistant pathogens into a state of high oxidative tension. Our data show that under drug pressure, KPC-2-carrying *K. pneumoniae* significantly down-regulates respiratory chain components (coenzyme Q) and lipid oxidative processes (acetylcarnitine) [22]. This “metabolic slowdown” likely serves to minimize the endogenous production of reactive oxygen species (ROS), which can synergize with antibiotics to cause irreversible cellular damage [23]. Concurrently, the activation of porphyrin metabolism and the depletion of glutathione levels indicate an intensified demand for antioxidant machinery to neutralize the drug-induced oxidative assault. The identification of NADH, FAD, and ADP as differentially abundant hubs is consistent with their established roles as universal energy and redox currencies. However, the coordinated fluctuation of these metabolites across both steady-state and stressed conditions suggests that KPC-2 carriage imposes a sustained drain on bioenergetic and redox pools, highlighting the physiological vulnerability of these central pathways under resistance-mediated metabolic burden [24].

### 4.5. Future Directions

In conclusion, this study provides a comprehensive systems-level view of the metabolic adaptations in KPC-2-producing *K. pneumoniae*. The transition from a growth-oriented baseline to a stress-adaptive metabolic mode reveals the intricate interplay between resistance genes and host physiology [25]. By highlighting the critical roles of membrane precursor synthesis, nucleotide gating, and redox management, we offer a new perspective on the survival mechanisms of high-risk pathogens [26]. Future research should focus on whether the metabolic intermediates identified here, such as specific purine derivatives or MEP pathway components, can be targeted by small-molecule inhibitors to resensitize these pathogens to conventional carbapenems. This represents a translational hypothesis emerging from our descriptive metabolomic dataset, rather than a conclusion supported by functional adjuvant screening data [11].

### 4.6. Limitations of the Study

Several limitations of this descriptive metabolomics investigation should be acknowledged. First, the use of a plasmid-based constitutive expression system precludes definitive attribution of metabolic shifts solely to KPC-2-specific physiological integration, as plasmid burden and selection pressure may contribute to the observed phenotype. Second, all metabolic interpretations are correlative; without functional validation through gene knockout complementation, isotopic flux analysis, or phenotypic rescue experiments, the proposed adaptive mechanisms remain hypothetical. Third, the findings are derived from a single genetic background; the generalizability of these metabolic signatures across diverse K. pneumoniae lineages (e.g., hypervirulent ST11 and ST258) requires independent validation. Finally, the central regulatory metabolites identified (NADH, FAD, UDP, and ADP) are universal metabolic hubs, and their differential abundance in this context, while informative, does not imply the establishment of new regulatory hierarchies.

## 5. Conclusions

This study provides a comprehensive characterization of the metabolic landscape in *Klebsiella pneumoniae* orchestrated by the expression of the KPC-2 resistance gene [27]. By employing high-resolution UHPLC-QE-Orbitrap-MS metabolomics, we have demonstrated that the presence of the *bla_KPC-2_* gene triggers a systematic and context-dependent reprogramming of bacterial cellular physiology [28]. Our findings indicate a profound systemic metabolic divergence, identifying 96 differential metabolites under steady-state conditions and 90 under antibiotic challenge. In the absence of drug pressure, KPC-2-carrying strains appear to undergo fitness trade-offs, potentially prioritizing cell membrane biogenesis and nucleotide metabolism at the expense of primary growth pathways, as evidenced by the marked depletion of essential amino acids—such as valine, isoleucine, and phenylalanine—and various carbohydrate intermediates [29]. Conversely, under antibiotic challenge, the bacteria transition into a specialized defensive mode characterized by the activation of porphyrin, purine, and aromatic amino acid metabolism. This adaptive shift is coupled with a strategic suppression of the respiratory chain and lipid oxidative processes, likely serving to mitigate endogenous oxidative stress. Network analysis further identified NADH, FAD, UDP, and ADP as the central regulatory nodes that sustain the metabolic and redox homeostasis of the resistant phenotype [30]. These results suggest that the metabolic threshold of nucleotide and energy pathways represents a significant physiological vulnerability. Much like our previous findings regarding the efficacy of exogenous adenosine in reversing *tet*(X3)-mediated resistance, the metabolic signatures identified here provide a hypothetical framework for the development of novel metabolic-based adjuvants to resensitize carbapenem-resistant pathogens [11]. In summary, our research elucidates the complex interplay between antibiotic resistance genes and host metabolism, providing a systemic perspective on the survival strategies of high-risk “superbugs” [31].

## Data Availability

The original contributions presented in the study are included in the article; further inquiries can be directed to the corresponding author.

## Abbreviations

The following abbreviations are used in this manuscript:

KPC-2	*Klebsiella pneumoniae* carbapenemase-2
UHPLC-QE-Orbitrap-MS	Ultra-high-performance liquid chromatography-hybrid quadrupole-Orbitrap mass spectrometry
OPLS-DA	Orthogonal partial least squares-discriminant analysis
ROS	Reactive oxygen species
NADH	Reduced nicotinamide adenine dinucleotide
FAD	Flavin adenine dinucleotide
UDP	Uridine diphosphate
dCMP	Deoxycytidine monophosphate
MEP	Non-mevalonate pathway/2-C-methyl-D-erythritol 4-phosphate pathway
HCA	Hierarchical Clustering Analysis
HILIC	Hydrophilic interaction liquid chromatography
RP	Reversed-phase
ESI	Electrospray ionization
